# Automatic Screening and Grading of Age-Related Macular Degeneration from Texture Analysis of Fundus Images

**DOI:** 10.1155/2016/5893601

**Published:** 2016-04-14

**Authors:** Thanh Vân Phan, Lama Seoud, Hadi Chakor, Farida Cheriet

**Affiliations:** ^1^Biomedical Engineering Institute of École Polytechnique de Montréal, Montréal, QC, Canada H3C 3A7; ^2^Université Libre de Bruxelles, 1050 Brussels, Belgium; ^3^Diagnos Inc., Brossard, QC, Canada J4Z 1A7; ^4^Department of Computer and Software Engineering of École Polytechnique de Montréal, Montréal, QC, Canada H3C 3A7

## Abstract

Age-related macular degeneration (AMD) is a disease which causes visual deficiency and irreversible blindness to the elderly. In this paper, an automatic classification method for AMD is proposed to perform robust and reproducible assessments in a telemedicine context. First, a study was carried out to highlight the most relevant features for AMD characterization based on texture, color, and visual context in fundus images. A support vector machine and a random forest were used to classify images according to the different AMD stages following the AREDS protocol and to evaluate the features' relevance. Experiments were conducted on a database of 279 fundus images coming from a telemedicine platform. The results demonstrate that local binary patterns in multiresolution are the most relevant for AMD classification, regardless of the classifier used. Depending on the classification task, our method achieves promising performances with areas under the ROC curve between 0.739 and 0.874 for screening and between 0.469 and 0.685 for grading. Moreover, the proposed automatic AMD classification system is robust with respect to image quality.

## 1. Introduction

**Figure 1:**
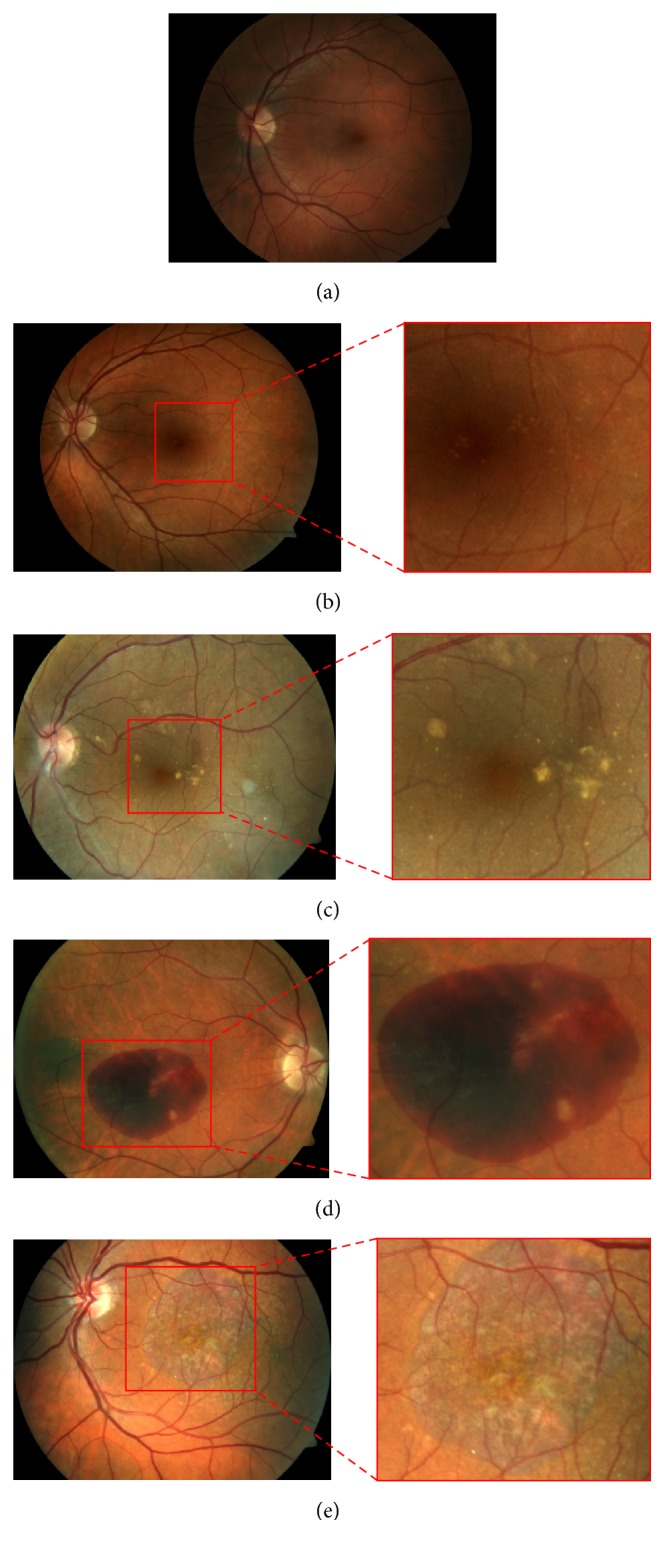
Images of macula area for different AMD categories: (a) healthy case in category {1}, (b) category {2} with hard drusen, (c) category {3} with soft drusen, and (d) category {4} with hemorrhages and (e) with geographic atrophy.

Age-related macular degeneration (AMD) is the main cause of visual deficiency and irreversible blindness in the elderly in Western countries [1]. It combines a variety of disorders affecting the macula. The early stage of AMD is asymptomatic, but small lesions, called drusen, can be revealed through examination of the retina. An increase in the size or number of drusen is a sign of the progression of the disease, leading eventually to the presence of hemorrhages (wet AMD) or to the development of geographic atrophy (late dry AMD). The Age-Related Eye Disease Study (AREDS) [2] proposed a simplified AMD clinical classification based on its stages. It comprises four categories which are illustrated in Figure 1: non-AMD {1}, mild {2}, moderate {3}, and advanced {4} AMD.

Currently, there is no approved treatment to recover from AMD. However, treatments to slow its progression exist and are different depending on the stage of the disease. These include prevention of oxidative damage, a treatment strategy based on supplements containing lutein, zeaxanthin, omega-3, vitamins C and E, and zinc, recommended for early stages [2, 3], while anti-VEGF therapy or surgical operations are used for more advanced stages [4].

With an aging population, there is urgent need for routine retinal examinations for early detection of AMD and for long-term follow-up strategies. Telescreening using fundus imaging has been extensively used for conditions like diabetic retinopathy [5, 6]. However, for AMD, it is still in its infancy. Combined with a telemedicine platform, automatic screening and grading from fundus images offer many inherent advantages. They allow clinicians to monitor susceptible individuals from an early age and to carry out preventive treatment.

Previous works focus mostly on dry AMD screening, based on the detection and quantification of drusen in fundus images [7]. The drusen segmentation techniques are categorized into methods based on either texture analysis, thresholding, clustering, edge detection, or template matching. A number of texture-based methods use Gabor filters [8], wavelet transform [9, 10], amplitude and frequency modulation [11, 12], statistical structure information [13], or gray-level cooccurrence matrix [14]. The segmentation is based on the response of drusen to the applied texture method, which is assumed to be different from the response of the background. Thresholding-based methods aim to find the appropriate threshold for separating the drusen from the background. This threshold can be set empirically [15] or automatically with Otsu's method [16]. Some image preprocessing is performed before thresholding using median or Gaussian filters [17], homomorphic filters [18], or morphological operations [19]. Methods based on clustering are used for AMD phenotype-genotype correlation [20] or for identifying AMD [21]. Drusen segmentation can also be achieved through edge detection by identifying abrupt intensity variations using gradient or Laplacian filters [22]. Finally, template matching methods use circular or Gaussian templates [23] to model and detect drusen using similarity measurements.

Other methods first detect drusen regions and a classification based on drusen features, using, for example, linear discriminant analysis, *k*-nearest neighbors, gentle boost, random forest, or support vector machine classifiers, is then performed for AMD screening or assessing the risk of progression to the advanced stage [24–26]. The results show good performance, comparable to trained human observers. However, drusen segmentation does not provide sufficient information for a complete AMD grading. In fact, in its advanced stages, drusen are often not observed, especially when there are large hemorrhages or atrophies. Moreover, even if these methods show high accuracy for hard drusen detection (up to 100%, with the best methods [12, 18]), the segmentation of soft drusen, which characterize the moderate cases, is highly challenging because of their diffuse shape [24, 25].

Other works focus on structures characterizing advanced stages, such as what is proposed in [27] which used machine learning for GA detection and segmentation. All these works on drusen and geographic atrophy detection and classification are useful for a deep analysis of specific stage of the disease. However, a combination of segmentation methods corresponding to each AMD structure may be computationally complex for screening and grading in a telemedicine context, where a large number of images must be analyzed.

To address these limitations, automatic AMD classification methods were performed based on directly computed image features, without prior segmentation. Kankanahalli et al. proposed a method based on visual context using SURF key points as features and a random forest (RF) for classification [28]. Different binary classifications such as {1&2} versus {3&4} or {1} versus {3} and a trinary classification ({1&2} versus {3} versus {4}) were considered to discriminate the moderate cases. Indeed, close attention to moderate cases is important because even though the patient still has adequate visual acuity, there is a considerable risk of progression to a more severe stage. The proposed method achieves a good accuracy (above 90%) for AMD severity classification. However, the evaluation was conducted on 2772 images out of 11344 available in the AREDS database (24.4% of the database), selected for their good quality. Since it was trained solely on good quality images, the classifier might not be as effective on images of lower quality. In a telemedicine context, in which the acquisition conditions are not always optimal, poor quality images are often encountered.

Prior preliminary studies [29, 30] conducted by our group for the evaluation of new features demonstrated promising results with local binary patterns (LBP) in multiresolution for AMD detection. However, the validation was conducted on small datasets and the different feature subsets were evaluated individually without considering any combination thereof. Moreover, these preliminary studies were limited to a binary classification aimed only at distinguishing images with and without AMD.

The aim of this paper is to propose and to evaluate an automatic approach for clinical AMD screening and grading in a telemedicine framework. Thus, it is important to develop a system which is robust to variable image quality. To do so, various features based on texture, color, and visual context were computed, evaluated for their relevance, and used to classify the images according to the different AREDS categories. The validation was performed on a highly heterogeneous set of 279 fundus images, acquired through an existing telemedicine platform (CARA for Computer-Aided Retina Analysis, Diagnos Inc., Canada). Additionally, the robustness of the classification system to poor quality images was evaluated.

The organization of the paper is as follows. In Section 2, the main steps of the proposed AMD classification method are described in detail. The experimental setup is explained in Section 3. The results are presented in Section 4, followed by a discussion in Section 5 and a conclusion in Section 6.

## 2. Materials and Methods

Fundus images acquired in a real screening context often show uneven illumination and poor contrast. To address these issues, a preprocessing step was required. Then, different features based on texture, color, and visual context were extracted to characterize the fundus image. Next, a classifier modeling step allowed us to measure the relevance of the features. Finally, two classifiers, SVM and RF, were tested on a database of 279 fundus images for performance assessment.

### 2.1. Image Preprocessing

Image normalization is required to correct the illumination drift introduced by the geometry of the eye and the bright flash of light used by the fundus camera. Contrast enhancement is also necessary to improve the information on details in the fundus images.

**Figure 2:**
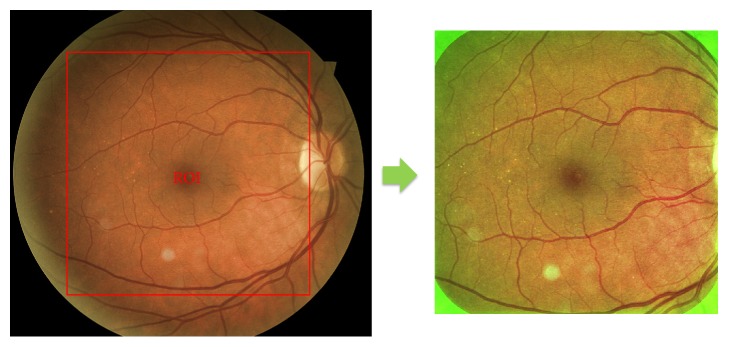
Preprocessing method: ROI corresponding to the square inscribed in the circle formed by the retina and the result of preprocessing with illumination normalization and contrast enhancement in green channel.

To perform these preprocessing steps, we used the same methodology as proposed in [28] for a fair comparison with their results. First, the region of interest (ROI) was defined as the square inscribed in the circle formed by the retina. Then, the green channel was extracted for preprocessing. A median filter with a kernel size of one-fourth the size of the ROI was applied in order to estimate the background illumination. The filtered image was then subtracted from the green channel of the original image. Finally, the green values were multiplied by 2 for contrast enhancement and shifted by the mean of their intensity range for visualization purposes (Figure 2).

### 2.2. Feature Extraction

Several features based on color, texture, and visual context were chosen because they proved to be effective in fundus image analysis. Color information is an intuitive feature, since AMD-related structures are characterized by specific colors. The texture and local gradient information also reflect the state of the retina. The image features considered in this study and their parameter settings are presented in the following subsections.

#### 2.2.1. Color Histograms

Blood vessels and lesions offer the highest contrast in the green channel. That is why most of the methods proposed in the literature for fundus image analysis focus solely on this channel. Still, even though the red channel is considered as saturated and with low contrast and the blue channel as very noisy in fundus images [31], all three color channels should be considered, especially to discriminate drusen from exudates, which are highly similar lesions but do not characterize the same disease [32]. In this study, the RGB and *L* ^*∗*^ *a* ^*∗*^ *b* ^*∗*^ spaces were used. In RGB, the red and blue channels provide additional information to the green one. The *L* ^*∗*^ *a* ^*∗*^ *b* ^*∗*^ space was also chosen because the luminance (lightness) and chrominance (colors) components are independent and color differences can be measured by a Euclidean distance.

We computed the 8-bin histograms for each channel from both color spaces as image features. The number of bins was set to 8 because there were no improvements in the results with a larger number of bins; thus we considered this sufficient for AMD classification.

#### 2.2.2. Local Binary Patterns (LBP) in Multiresolution

To obtain the multiresolution information, a Lemarié wavelet transform was used with four levels of decomposition. For each level, an approximation coefficient and three detail coefficients were computed, containing, respectively, the low resolution image (original size divided by two) and the high resolution details in the horizontal, vertical, and diagonal directions. From the original image and the 16 coefficient images, textural information was extracted using LBP. This consisted in measuring the occurrence of local texture primitives, such as corners or edges. To do so, the LBP [33] was computed for each pixel of gray value *g*_*c*_ in a neighborhood of radius *R* and *P* neighbors with gray values *g*_*p*_:

(1) LBPP,R=∑p=0P−1sgp−gc2P,With  sx=1,if  x≥0,0,Otherwise.

In this study, the parameters were empirically set to *R* = 1 and *P* = 8. The magnitude component of the LBP [34] was also computed from the absolute differences of gray intensity between the central pixel and its neighbors *m*_*p*_ = |*g*_*p*_ − *g*_*c*_|:

(2) LBPMP,R=∑p=0P−1tmp,c2P,With  tx,c=1,if  x≥c,0,Otherwise.

The threshold *c* was set to the image mean value.

From the sign and magnitude components of LBP, two histograms were computed by measuring the occurrence of the different patterns in the image. For each RGB color channel, LBP were computed and generated a vector of 2006 features.

#### 2.2.3. Histograms of Oriented Gradients (HOG)

The histogram of oriented gradients is a feature generally used for edge detection [35], but it also contains local directional information which can be used for classification.

The horizontal and vertical gradients were computed by applying a 1D point-centered derivative kernel [−1 0 1] to the color image. Then, local histograms of the four main directions were constructed by dividing the RGB color image into 16 × 16 cells, with 2 × 2 cells for block normalization. The constructed vector contained 3600 features.

#### 2.2.4. SURF Key Points

Starting from the hypothesis that all AMD manifestations (drusen and other lesions) were represented in the subset of images presenting AMD, SURF key points were computed on that subset of images, previously converted into *L* ^*∗*^ *a* ^*∗*^ *b* ^*∗*^. The key points were detected using ten octaves, three layers per octave, and a Hessian threshold of 600. Using the SURF features (sign of Laplacian, orientation, scale of detection, and strength of detected feature), a *K*-means clustering selected centroids on which the vocabulary was based to construct the features vector. For binary classifications, *K* was set to 100, while for multiclass classifications, *K* was set to 300. All parameters used to compute the SURF key points and to construct the vocabulary were set empirically. Once the vocabulary was established, a histogram was constructed by measuring the occurrence of key points depending on the nearest centroid. These features are implemented as proposed in [28] with unchanged parameters values.

### 2.3. Dimensionality Reduction and Features Importance

On one hand, a dimensionality reduction is necessary to avoid overfitting. Indeed, we have 6018 LBP features (2006 on each channel), 96 color histograms features, 3600 HOG features, and 100 or 300 SURF features. Considering the size of our dataset, a dimensionality reduction step is required before training a classifier. On the other hand, we believe that some of the features used might be more relevant than others in the discrimination between AMD stages. Thus, in order to evaluate features relevance and to select optimal subsets of features for classification, we used two approaches.

#### 2.3.1. Fisher's Criterion

We determined the feature's relevance using the approach based on the Fisher criterion, which must be maximized [36]. This criterion measures the divergence between two classes *i* and *j* based on the estimate of their means *μ* and standard deviations *σ* when they are projected on the feature axis *F*:

(3) $$\begin{array}{c} D\left(\;F\;\right)=\frac{{\left(\mu_{i}-\mu_{j}\right)}^{2}}{\sigma_{i}^{2}+\sigma_{j}^{2}}. \end{array}$$

The maximum number of features for classifier modeling was set to one-tenth the number of training samples [37]. The final number of features retained was determined based on the best SVM performance obtained by varying the number of features and testing on validation samples.

#### 2.3.2. Features Importance Using Gini Index

We also used the features' relevance assessment performed in random forest training [38]. We considered the mean decrease in Gini index to measure the features' relevance. This parameter measures the loss in Gini index on the out-of-bag samples when the feature is removed or permuted. The larger the decrease is, the more relevant the feature is. In this experiment, we used 3000 trees and we set the number of features to be selected at each node to 25 to ensure that all features are considered in the model to evaluate its importance.

### 2.4. Classifier Modeling

Two different classifiers were used in this study to verify if the choice of classifier has a significant impact on the results: a support vector machine (SVM) and a random forest (RF).

#### 2.4.1. Support Vector Machine (SVM)

The training of an SVM consists in finding the decision boundary that maximizes the margin that is the space between the elements nearest to the boundary [39].

In this study, a Gaussian kernel was chosen for the SVM because it is more efficient for systems with complex separations than a linear classifier. In addition, SVMs are useful for systems with a small number of samples because only the elements near the boundary, that is, the support vectors, contribute to the SVM modeling. For classifier modeling, the parameters to be set are *γ*, the parameter of the Gaussian kernel, and *C*, the number of elements accepted in the margin. These parameters were set according to a performance assessment using a grid search strategy with 10-fold cross-validation to find the best pair of values in gamma = [0.001,0.01,0.1,1, 10] and *C* = [1,10,50,100].

To consider more than two classes, we used the one-against-all approach. In the training phase, one SVM per class is constructed to discriminate the samples of that class from all the others. The label of a new observation is then determined by the SVM classifier that returns the highest value.

#### 2.4.2. Random Forest (RF)

The training of an RF consists in constructing decision trees, using randomly selected training samples and features. Then, the classification of new samples is determined by aggregating the votes of the different trees [40]. This method is quite simple to use since only two parameters need to be set: the number of features in the random subset at each tree node and the number of trees in the forest [41]. The first parameter was set to the square root of the total number of features. The second parameter was set to 1,000 decision trees for binary classification and 2,500 decision trees for multiclass classification, such as what is proposed in [28].

## 3. Experimental Setup

### 3.1. Materials

The validation was conducted on a database of 279 images, all provided by Diagnos Inc. (Brossard, QC, Canada) using their telemedicine platform. The images were collected from clients in the United Arab Emirates, Mexico, Poland, India, and Canada. The devices used for the acquisitions are different models of Zeiss, DRS, Topcon, and Canon retinal cameras. All the images are in JPEG compressed 10 : 1 format and acquired with a 45° field-of-view. Depending on the camera used, the size of the images varies between 1,400, 2,200, and 3,240 pixels along the diameter of the retinal images (circular imaged region excluding black background).

**Figure 3:**
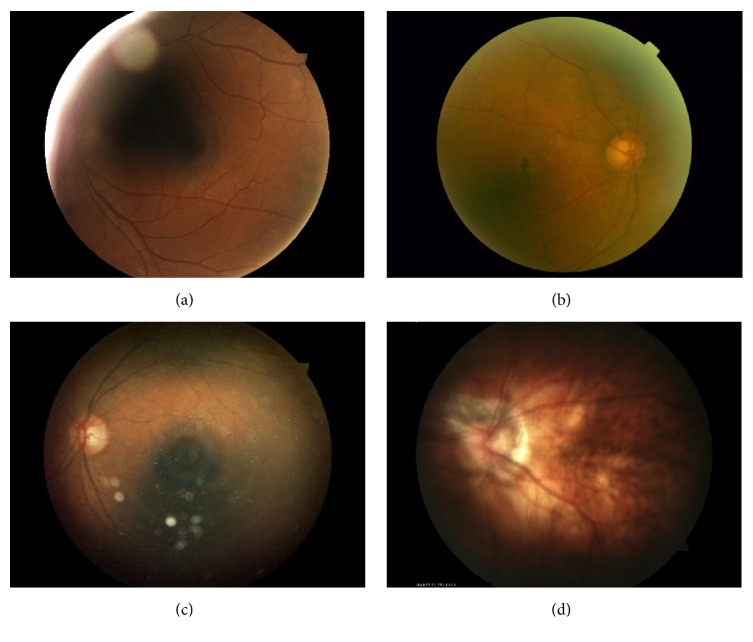
Examples of poor quality images: (a) shadows and intense reflections, (b) haze, (c) arc and specular reflections, and (d) blur.

Depending on the acquisition conditions, the images vary in terms of resolution and illumination both of which affect the image quality [42]. Different artefacts, illustrated in Figure 3, can be encountered in fundus photography: shadows, intense reflections, specular reflections, blur, haze, or arcs. In this study, we used an automatic image quality assessment method described in [43]. The algorithm determined if an image is of good or poor quality based on its measured color distribution and sharpness.

Two human graders were instructed to label the images into one of the four AREDS categories. The first grader (Grader A) is an ophthalmologist with 10 years of experience working on fundus images. He has expertise in AREDS classification. The second grader (Grader B) has 2 years of experience working on fundus images and was trained to classify fundus images following the simplified AMD classification proposed by the AREDS.

The number of images in each AREDS category (as labeled by Grader B) and their distribution according to quality level are shown in Table 1.

### 3.2. Experiments

#### 3.2.1. Dimensionality Reduction and Features Relevance

To reduce the feature space dimension, we used, on one hand, the feature selection based on Fisher's criterion and, on the other hand, the features' relevance assessment based on mean decrease of Gini index for each classification task. Then, we counted the number of selected features in each feature category to highlight the most relevant features for AMD classification.

#### 3.2.2. Performance Assessment for Screening

To assess the performance of our method for AMD screening, we evaluated several binary classification tasks, using a 10-fold cross-validation approach. This consisted in taking one-tenth of the dataset as a testing set, and the rest was used to train the classifier. The prediction result from this classification was kept and the process was repeated for all the elements. Receiver Operating Characteristic (ROC) curves were obtained by varying the threshold on the probabilities given by both classifiers (SVM and RF) and by reporting the sensitivity and specificity corresponding to this threshold. The corresponding areas under the ROC curves (AUC) were then computed. We also tested statistically how the results are significantly different from a random classifier [44].

#### 3.2.3. Performance Assessment for Grading

In the same way as for screening, the performance for AMD grading was assessed using a 10-fold cross-validation approach for multiclass classifications using SVM and RF. The results were then compared to the intergrader variability. These results are reported using the confusion matrix, the classification accuracy (number of elements that are well classified), and the weighted Kappa coefficient [45].

#### 3.2.4. Robustness to Image Quality

Selecting good quality images to train a classification system does not guarantee its efficiency for processing images of variable quality, for example, in a telemedicine context. To evaluate and to compare the robustness to variations in image quality, an assessment using only good quality images for training and poor quality images for testing was performed. In this experiment, we also performed SVM and RF training and testing using only the SURF features as proposed in [28] for ends of comparison.

Our overall approach for performance assessment aimed at determining the best solution for robust AMD classification.

## 4. Results

### 4.1. Features Relevance

The features relevance was evaluated for screening and grading to highlight the most relevant features for an automatic classification following the AREDS criteria.  Table 2 shows the number of features selected according to Fisher's criterion and Gini index. For both features selection methods, LBP features are the most selected for any classification tasks, especially LBP features computed in green channel. These features are the most relevant for AMD classification.

It is also to be noted that SURF features are never selected by neither the Fisher based method nor the RF Gini method. It appears that these features are not the most relevant to discriminate between the different AMD stages.

### 4.2. Performance Assessment for Screening

**Figure 4:**
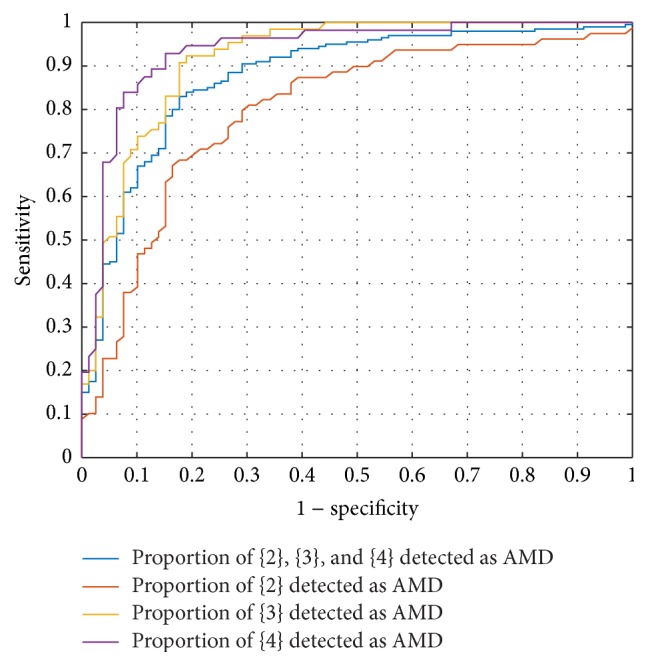
Screening performance for {1} versus {2&3&4} using SVM classifier and features selected using RF Gini.

The AMD classification for screening {1} versus {2&3&4} was assessed for both classifiers, with and without a features selection step (see Table 3). The best results were obtained with the features selected based on Gini index, with an AUC of 87.7% for SVM and an AUC of 86.9% for RF. In Figure 4, the specificity and sensitivity corresponding to mild {3}, moderate {3}, and severe {4} are presented along with the ROC curve. It shows that cases in categories {3} and {4} are better detected as AMD than category {2}.

In light of these results, we decided to assess the classification {1&2} versus {3&4}, since a large proportion of cases in category {2} were considered as non-AMD. This classification task corresponds to distinguishing cases that require treatment (moderate and advanced cases) from cases that are not at risk (healthy and mild cases). The performance is better than previously mentioned with an AUC of 89.9% for SVM and an AUC of 89.8% for RF.

### 4.3. Performance Assessment for Grading

The results of performance assessment for grading are shown in Table 4. For each classification task, the best results were obtained with the features selected based on Gini index and the SVM classifier. For the automatic classification according to AREDS protocol ({1} versus {2} versus {3} versus {4}), the method achieved an accuracy of 62.7%. Accuracies of 75.6% and 72.4% were obtained, respectively, for {1&2} versus {3} versus {4} and for {1} versus {2&3} versus {4}. The results demonstrate that the classification gives better performance when the number of categories to classify is lower.

Table 5 presents the confusion for {1} versus {2} versus {3} versus {4} using features selected by Gini index. Most of the misclassifications happen between categories {1} and {2}. That explains why the performance was better when we considered {1&2} as one category. We also compared the results with intergrader variability. The latter was assessed on a subset of 176 images annotated by both Graders A and B and measured with weighted Kappa coefficient. The results (see Table 5) showed a weighted Kappa coefficient of 73,6%, which corresponds to a substantial agreement between graders [45]. The automatic method does not reach a performance on the same order as the intergrader variability.

However, we can notice that, even for graders, most disagreements happen between classes {1} and {2} and between {2} and {3}.

From these results, we also tested a classification in two steps. First, we classified all images into three categories {1&2}, {3}, and {4}, since trinary classification gives better results. Then, the cases in {1&2} are classified into {1} and {2}. The results are shown in Table 6 and, indeed, improved with a weighted Kappa of 66.2% for SVM and of 61.0% for RF, which corresponds to a substantial agreement. For the SVM classifier, the weighted Kappa is in the 95% confidence interval of the intergrader Kappa which means that there is no significant difference between the performance of the automatic SVM classifier and Grader B, when compared to Grader A.

### 4.4. Robustness to Variable Image Quality

The robustness was assessed by measuring the performance of the system when trained with only good quality images and tested on poor quality images. We compared our results with the method proposed in [28] which is based solely on the SURF features as described in Section 2.2.4.  Table 7 shows the robustness assessment for AMD screening. The resulting AUCs are in the same range as in the 10-fold cross-validation on the whole dataset (Table 4).  Table 8 shows the robustness assessment for AMD grading. Here, the classification accuracy decreases compared to the assessment by 10-fold cross-validation on the whole dataset (Table 5), yielding accuracies of 0.207–0.557 with SVM and 0.393–0.693 with RF.

## 5. Discussion

The main purpose of this paper was to propose an automatic system for clinical AMD screening and grading in a telemedicine framework and to evaluate its performance. This was achieved through a comparative study of different image features mainly based on texture, color, and visual context.

The experiments revealed that the best results for AMD screening and grading were obtained with LBP in multiresolution applied to the green channel. These features were considered as the most relevant for AMD classification and were favored by the Fisher criterion and Gini index. The present work confirms that these features are robust with respect to image quality, as suggested in our prior studies [29, 30], and extends those results from AMD detection to AMD severity classification. Even with small learning samples, the systems using SVM classifier and features selected by Gini index achieved AUCs between 0.877 and 0.899 for AMD screening, which is especially good considering the large proportion of poor quality images (50.2% of the database). Our best result for AMD grading was an accuracy of 75.6% for the trinary classification task {1&2} versus {3} versus {4}. The automatic grading following AREDS protocol was in the same order as intergrader variability while using SVM and features selected based on Gini index.

LBP is a powerful tool for characterizing the texture and that is why these features are the most suitable for this application. First, a local characterization of the texture is more effective than a global feature such as color histograms. Then, LBP measures the proportions of the different uniform patterns contained in the image (such as edges, borders, or points), which seem to be more informative than the local gradients computed in HOG or the SURF key point features. In fact, these latter features seem to be less robust to poor quality images, since they are based on detecting local maxima which can be sensitive to noise. Thus, LBP are the most reliable features taking into account the types of structures characterizing AMD images at different severity degrees. Finally, the multiresolution approach helps us to characterize the stage of the disease by identifying lesions at different scales. Indeed, a lesion detected at high resolution could correspond to large drusen or an atrophy, both being related to more advanced AMD stages.

We have proposed a method that is adapted to a real telemedicine context. This means that we processed images from variable quality levels, coming from different locations and different cameras, whereas major studies on AMD in the literature have used homogeneous datasets. Furthermore, our results compare well against those of other methods. For AMD screening, a study carried out in [24] aimed to evaluate if cases were at low or high risk to progress to an advanced stage, based on drusen segmentation. Their system achieved a Kappa coefficient of 0.760–0.765. This is similar to our classification performance for {1&2} versus {3&4}, which obtained AUCs of 0.899. Nevertheless, it is difficult to compare these different methods one on one since there is no publicly available database for AMD grading containing fundus images labeled according to AREDS protocol.

For AMD grading, the method proposed in [28] reports an accuracy of 91.5% for classifying {1&2} versus {3} versus {4} on selected images of good quality. Our method achieved an accuracy of 75.6%, which is significantly lower; however all images were processed including images of poor quality. To support that furthermore, the experiment on robustness to image quality clearly demonstrates that AMD screening and grading using SURF features as proposed in [28] is not applicable in a telemedicine setting where image quality is not always guaranteed.

Our method demonstrates considerable robustness with respect to image quality. In a telemedicine context, where acquisition conditions are not strictly controlled, to only select good quality images is not adequate for AMD evaluation because we want a maximum of cases to be handled. To demonstrate the robustness to image quality, we assessed the classification systems performance by training them on good quality images and testing them on poor quality ones. Our system still performed well, presenting results of the same order as the ones obtained in the leave-one-out cross-validation.

In regard to the classification tasks, it is recommended to use the classification {1&2} versus {3&4} for AMD screening, which presented a better result using our method. The clinical rationale for this classification is to distinguish cases that need to be treated from those that are not at risk. We can notice that our method tends to consider a certain proportion of category {2} cases as non-AMD. For grading, a better classification performance is obtained for a two-step classification, starting with {1&2} versus {3} versus {4} classification and then performing a {1} versus {2} classification.

Our database contained a relatively small number of samples in each category. This may be the reason why a good performance for grading could not be demonstrated in this study. Moreover, even the human graders had some difficulty agreeing on the database's labeling, with an intergrader weighted Kappa of 0.736. A validation on a larger database could improve the grading results.

Future work will focus on the preprocessing step. In fact, in this study, we used a preprocessing procedure introduced in [28] for ends of comparison. Nevertheless, several improvements could be made to it. The background illumination was estimated with a median filter, but the convolution with a high resolution image has a large computational cost. This aspect could be improved by using spectral filtering instead. Also, our previous work demonstrated that a local analysis focused on the macular area can improve the system performance. Indeed, features of AMD are mainly located in this area. This idea could be further explored by using an automatic detection of the macular region based on the detection of the fovea and the radius of the optic disc.

## 6. Conclusion

We have developed and validated an automatic clinical classification system for AMD screening and grading in a telemedicine context. The validation of our method reveals promising results in terms of robustness to image quality and accuracy for different AMD severity classification tasks. Our experimental results highlight the discriminating strength of LBP features over other tested features, whether the classifier is an RF or an SVM. Further validation must be conducted on a database containing more samples in each category in order to confirm these results. Nevertheless, the proposed approach represents an important step toward providing a reliable AMD diagnostic tool for patient monitoring and for clinical trials. Early AMD detection can facilitate timely access to treatment and consequently improve the therapeutic outcome.

## Figures and Tables

**Table 1:** Number of images in each AREDS category and for each image quality level.

Category	{1} Non-AMD	{2} Early	{3} Moderate	{4} Advanced
Good quality	50	43	24	22
Poor quality	29	36	41	34

**Table 2:** Number of selected features per category.

Classifications	Features selection	Features categories						
		LBP red	LBP green	LBP blue	RGB hist.	Lab hist.	HoG	SURF
All	None	2006	2006	2006	48	48	3600	100
1_234	Fisher	4	4	0	0	0	0	0
	RF Gini	92	114	27	1	1	31	0
12_34	Fisher	2	6	0	0	0	0	0
	RF Gini	63	79	18	0	0	17	0
12_3_4	Fisher	0	8	0	0	0	0	0
	RF Gini	74	94	23	1	1	23	0
1_23_4	Fisher	0	5	0	0	0	0	0
	RF Gini	82	106	25	1	1	29	0
1_2_3_4	Fisher	0	7	0	0	0	0	0
	RF Gini	92	114	29	1	1	31	0

**Table 3:** Performance assessment (AUC) for screening.

Classifier		SVM			RF		
Features selection		None	Fisher	Gini	None	Fisher	Gini
1_234	AUC	0.494	0.743^*∗*^	0.877^*∗*^	0.791^*∗*^	0.812^*∗*^	0.869^*∗*^
12_34	AUC	0.491	0.879^*∗*^	0.899^*∗*^	0.867^*∗*^	0.843^*∗*^	0.898^*∗*^

*∗*: statistically different from random classifier (0.5 not included in 95% CI of AUC).

**Table 4:** Performance assessment (accuracy) for grading.

Classifier		SVM			RF		
Features selection		None	Fisher	Gini	None	Fisher	Gini
12_3_4	Acc.	0.563	0.667	0.756	0.688	0.695	0.742
1_23_4	Acc.	0.516	0.581	0.724	0.642	0.613	0.699
1_2_3_4	Acc.	0.280	0.477	62.7	0.513	0.484	0.617

**Table 5:** Confusion matrix in percentage for grading ({1} versus {2} versus {3} versus {4}).

%	SVM (Gini)				RF (Gini)				Grader B			
Nb img	279				279				176			
Grader A	1	2	3	4	1	2	3	4	1	2	3	4

1	20.1	6.8	1.1	0.4	19.7	6.5	1.4	0.7	31.2	9.5	0.6	0.0
2	6.5	15.8	4.7	1.4	7.2	16.5	2.9	1.8	4.5	19.3	6.2	0.6
3	1.4	4.7	13.3	3.9	2.2	5.7	13.3	2.1	0.0	3.4	7.4	2.8
4	0.7	0.7	5.0	13.6	0.7	2.2	5.0	12.2	0.0	1.1	1.1	13.1

Accuracy	62.7				61.6				71.5			

Weighted *K* (95% CI)	63.7 (57.3–70.2)				59.4 (52.3–66.5)				73.6 (66.1–80.2)			
	Substantial				Moderate				Substantial			

**Table 6:** Confusion matrix in percentage for grading in two steps ({1&2} versus {3} versus {4} and then {1} versus {2}).

%	SVM (Gini)				RF (Gini)				Grader B			
Nb img	279				279				176			
Grader A	1	2	3	4	1	2	3	4	1	2	3	4

1	22.6	4.3	1.1	0.3	21.9	5.0	0.7	0.7	31.2	9.5	0.6	0.0
2	4.3	18.3	4.3	1.4	4.7	19.7	2.5	1.4	4.5	19.3	6.2	0.6
3	1.8	4.7	12.2	4.6	3.6	7.1	10.0	2.5	0.0	3.4	7.4	2.8
4	0.7	1.1	5.0	13.3	1.1	1.8	4.7	12.5	0.0	1.1	1.1	13.1

Accuracy	66.3				64.2				71.5			

Weighted *K* (95% CI)	66.2 (59.7–72.6)				61.0 (53.8–68.1)				73.6 (66.1–80.2)			
	Substantial				Substantial				Substantial			

**Table 7:** Quality robustness assessment (AUC) for screening.

Classifier		SVM				RF			
Features selection		None	SURF [28]	Fisher	RF Gini	None	SURF [28]	Fisher	RF Gini
1_234	AUC	0.500	0.500	0.588	0.874^*∗*^	0.797^*∗*^	0.436	0.807^*∗*^	0.889^*∗*^
12_34	AUC	0.500	0.530	0.882^*∗*^	0.812^*∗*^	0.819^*∗*^	0.472	0.875^*∗*^	0.816^*∗*^

*∗*: statistically different from random classifier (0.5 not included in 95% CI of AUC).

**Table 8:** Quality robustness assessment (accuracy) for grading.

Classifier		SVM				RF			
Features selection		None	SURF [28]	Fisher	RF Gini	None	SURF [28]	Fisher	RF Gini
12_3_4	Acc.	0.466	0.464	0.529	0.557	0.607	0.493	0.571	0.586
1_23_4	Acc.	0.550	0.550	0.550	0.550	0.643	0.329	0.557	0.693
1_2_3_4	Acc.	0.207	0.300	0.450	0.507	0.486	0.350	0.393	0.521

## References

[1] Kasuga D. T., Chen Y., Zhang K. (2011). Genetics of age-related degeneration. *Age-Related Macular Degeneration Diagnosis and Treatment*.

[2] Age-Related Eye Disease Study Research Group (AREDS) (2005). The age-related eye disease study severity scale for age-related macular degeneration. *Archives of Ophthalmology*.

[3] Meleth A. D., Raiji V. R., Krishnadev N., Chew E. Y., Ho A. C., Regillo C. D. (2011). Therapy of nonexudative age-related macular degeneration. *Age-Related Macular Degeneration Diagnosis and Treatment*.

[4] Penha F. M., Rosenfeld P. J., Ho A. C., Regillo C. D. (2011). Management of neovascular AMD. *Age-Related Macular Degeneration Diagnosis and Treatment*.

[5] Oliveira C. M., Cristóvão L. M., Ribeiro M. L., Faria Abreu J. R. (2011). Improved automated screening of diabetic retinopathy. *Ophthalmologica*.

[6] Seoud L., Hurtut T., Chelbi J., Cheriet F., Langlois J. M. (2016). Red lesion detection using dynamic shape features for diabetic retinopathy screening. *IEEE Transactions on Medical Imaging*.

[7] Kanagasingam Y., Bhuiyan A., Abràmoff M. D., Smith R. T., Goldschmidt L., Wong T. Y. (2014). Progress on retinal image analysis for age related macular degeneration. *Progress in Retinal and Eye Research*.

[8] Parvathi S. S., Devi N. Automatic drusen detection from colour retinal images.

[9] Brandon L., Hoover A. (2003). Drusen detection in a retinal image using multi-level analysis. *Medical Image Computing and Computer-Assisted Intervention—MICCAI*.

[10] Priya R., Aruna P. Automated diagnosis of Age-related macular degeneration from color retinal fundus images.

[11] Barriga E. S., Murray V., Agurto C. Multi-scale AM-FM for lesion phenotyping on age-related macular degeneration.

[12] Agurto C., Barriga E., Murray V. (2011). Automatic detection of diabetic retinopathy and age-related macular degeneration in digital fundus images. *Retina*.

[13] Köse C., Şevik U., Gençalioğlu O., İkibaş C., Kayıkıçıoğlu T. (2010). A statistical segmentation method for measuring age-related macular degeneration in retinal fundus images. *Journal of Medical Systems*.

[14] Prasath A. R., Ramya M. M. (2015). Detection of macular drusen based on texture descriptors. *Research Journal of Information Technology*.

[15] Morgan W. H., Cooper R. L., Constable I. J., Eikelboom R. H. (1994). Automated extraction and quantification of macular drusen from fundal photographs. *Australian and New Zealand Journal of Ophthalmology*.

[16] Smith R. T., Chan J. K., Nagasaki T. (2005). Automated detection of macular drusen using geometric background leveling and threshold selection. *Archives of Ophthalmology*.

[17] Soliz P., Wilson M. P., Nemeth S. C., Nguyen P. Computer-aided methods for quantitative assessment of longitudinal changes in retinal images presenting with maculopathy.

[18] Rapantzikos K., Zervakis M., Balas K. (2003). Detection and segmentation of drusen deposits on human retina: potential in the diagnosis of age-related macular degeneration. *Medical Image Analysis*.

[19] Liang Z., Wong D. W. K., Liu J., Chan K. L., Wong T. Y. Towards automatic detection of age-related macular degeneration in retinal fundus images.

[20] Quellec G., Russell S. R., Abramoff M. D. (2011). Optimal filter framework for automated, instantaneous detection of lesions in retinal images. *IEEE Transactions on Medical Imaging*.

[21] Hanafi M., Hijazi A., Coenen F., Zheng Y. Retinal image classification for the screening of age-related macular degeneration.

[22] Mora A. D., Vieira P. M., Manivannan A., Fonseca J. M. (2011). Automated drusen detection in retinal images using analytical modelling algorithms. *BioMedical Engineering Online*.

[23] Remeseiro B., Barreira N., Calvo D., Ortega M., Penedo M. G. (2009). Automatic drusen detection from digital retinal images: AMD prevention. *Computed Aided Systems Theory-EUROCAST*.

[24] Van Grinsven M. J. J. P., Lechanteur Y. T. E., Van De Ven J. P. H., Van Ginneken B., Theelen T., Sánchez C. I. Automatic age-related macular degeneration detection and staging.

[25] Akram M. U., Mujtaba S., Tariq A. Automated drusen segmentation in fundus images for diagnosing age related macular degeneration.

[26] Sundaresan V., Ram K., Selvaraj K., Joshi N., Sivaprakasam M. Adaptative super-candidate based approach for detection and classification of drusen retinal fundus images.

[27] Feeny A. K., Tadarati M., Freund D. E., Bressler N. M., Burlina P. (2015). Automated segmentation of geographic atrophy of the retinal epithelium via random forests in AREDS color fundus images. *Computers in Biology and Medicine*.

[28] Kankanahalli S., Burlina P. M., Wolfson Y., Freund D. E., Bressler N. M. (2013). Automated classification of severity of age-related macular degeneration from fundus photographs. *Investigative Ophthalmology & Visual Science*.

[29] Garnier M., Hurtut T., Tahar H. B., Cheriet F. Automatic multiresolution age-related macular degeneration detection from fundus images.

[30] Phan T. V., Seoud L., Cheriet F. Towards an automatic classification of age-related macular degeneration.

[31] Walter T., Massin P., Erginay A., Ordonez R., Jeulin C., Klein J.-C. (2007). Automatic detection of microaneurysms in color fundus images. *Medical Image Analysis*.

[32] van Grinsven M. J. J. P., Chakravarty A., Sivaswamy J., Theelen T., van Ginneken B., Sanchez C. I. A Bag of Words approach for discriminating between retinal images containing exudates or drusen.

[33] Ojala T., Pietikäinen M., Mäenpää T. (2002). Multiresolution gray-scale and rotation invariant texture classification with local binary patterns. *IEEE Transactions on Pattern Analysis and Machine Intelligence*.

[34] Guo Z., Zhang L., Zhang D. (2010). A completed modeling of local binary pattern operator for texture classification. *IEEE Transactions on Image Processing*.

[35] Dalal N., Triggs B. Histograms of oriented gradients for human detection.

[36] Duda R. O., Hart P. E., Stork D. G. (2009). Maximum-likelihood and Bayesian parameters estimation. *Pattern Classification*.

[37] Webb A. R. (2003). *Statistical Pattern Recognition*.

[38] Breiman L. (2002). *Manual on Setting Up, Using and Understanding Random Forests V3.1*.

[39] Steinwart I., Christmann A. (2008). *Support Vector Machines*.

[40] Breiman L. (2001). Random forests. *Machine Learning*.

[41] Liaw A., Wiener M. (2002). Classification and regression by random forest. *R News*.

[42] Bartling H., Wanger P., Martin L. (2009). Automated quality evaluation of digital fundus photographs. *Acta Ophthalmologica*.

[43] Fasih M., Langlois J. M. P., Tahar H. B., Cheriet F. Retinal image quality assessment using generic features.

[44] DeLong E. R., DeLong D. M., Clarke-Pearson D. L. (1988). Comparing the areas under two or more correlated receiver operating characteristic curves: a nonparametric approach. *Biometrics*.

[45] Viera A. J., Garrett J. M. (2005). Understanding interobserver agreement: the kappa statistic. *Family Medicine*.

